# Comprehensive comparison of graph based multiple protein sequence alignment strategies

**DOI:** 10.1186/1471-2105-13-64

**Published:** 2012-04-29

**Authors:** Ilya Plyusnin, Liisa Holm

**Affiliations:** 1 Institute of Biotechnology, University of Helsinki, P.O. Box 56, Viikinkaari 5, FIN-00014 Helsinki, Finland; 2 Department of Biological and Environmental Sciences, University of Helsinki, P.O.Box 56, Viikinkaari 5, FIN-00014 Helsinki, Finland

## Abstract

**Background:**

Alignment of protein sequences (MPSA) is the starting point for a multitude of applications in molecular biology. Here, we present a novel MPSA program based on the SeqAn sequence alignment library. Our implementation has a strict modular structure, which allows to swap different components of the alignment process and, thus, to investigate their contribution to the alignment quality and computation time. We systematically varied information sources, guiding trees, score transformations and iterative refinement options, and evaluated the resulting alignments on BAliBASE and SABmark.

**Results:**

Our results indicate the optimal alignment strategy based on the choices compared. First, we show that pairwise global and local alignments contain sufficient information to construct a high quality multiple alignment. Second, single linkage clustering is almost invariably the best algorithm to build a guiding tree for progressive alignment. Third, triplet library extension, with introduction of new edges, is the most efficient consistency transformation of those compared. Alternatively, one can apply tree dependent partitioning as a post processing step, which was shown to be comparable with the best consistency transformation in both time and accuracy. Finally, propagating information beyond four transitive links introduces more noise than signal.

**Conclusions:**

This is the first time multiple protein alignment strategies are comprehensively and clearly compared using a single implementation platform. In particular, we showed which of the existing consistency transformations and iterative refinement techniques are the most valid. Our implementation is freely available at http://ekhidna.biocenter.helsinki.fi/MMSA and as a supplementary file attached to this article (see Additional file 1).

## Background

A variety of methods used by modern molecular biology such as structural modeling, function annotation, phylogenetic analysis and similarity searches are based on multiple protein sequence alignments (MPSA). Correct MPSA arranges in one column position amino acids that share a common ancestor or are functionally/structurally equivalent. MPSA provides position-specific information on evolutionary conserved characters, correlation between characters and their distribution. These features can be used in many further applications for which the quality of MPSA is, therefore, crucial [1]. Traditionally, the quality of the alignment is evaluated using a scoring function based on gap penalties and a substitution matrix. When two sequences are aligned, an exact solution can be found using dynamic programming [2,3]. When many sequences are aligned, the exact solution is computationally expensive and becomes intractable for more than a few sequences [4]. The majority of methods avoid exponential scaling of alignment problem by employing various greedy heuristics, including a widely used progressive alignment technique [5]. Classic progressive methods such as ClustalW [6] are fast and can produce reasonable results for sequences that are sufficiently similar (e.g., show identity higher then 40%) but become impractical in the so called twilight zone (e.g., identity below 30%) [1,7]. A vast amount of research has been conducted in order to improve alignment quality for distant sequences. Many methods that have succeeded in this task are based on modifying the scoring function. We use a unified graph framework to compare different scoring functions.

In this article we shall use the term "alignment graph" as it is defined in the graph-based alignment algorithm by Rausch et al. [8] and the corresponding implementation in the SeqAn sequence alignment library [9]. The alignment graph represents residues of aligned sequences by vertices and various signals on which the alignment is based, such as residue substitutions from the substitution model, structural links etc., by edges connecting the vertices. This provides a flexible model for scoring: any type of information can be introduced by adding edges or weights to the existing edges, while certain transformations of the graph help to avoid local minima during the optimization step.

The separation of the input signal for the alignment from the process that produces the alignment was pioneered by T-Coffee [10]. This program starts by compiling a library of local and global pairwise alignments, which equals to populating the alignment graph with the corresponding edges. T-Coffee was then extended to include structural information [11] and to combine alignments produced by other methods into a consensus alignment [12]. Another example is MaxFlow [13], which initializes the alignment graph with information from PSI-BLAST searches. We note that, in spite of these implementations, the contribution of various information sources to the quality of MPSA is yet to be analyzed. Here, we supply this need by presenting a clear comparison of sequence based information sources.

Various consistency transformations have been shown to effectively improve alignments of distant sequences [10,14,15]. The idea is to transform scores used to align pairs of sequences to be consistent between different pairs. For example, for three sequences A, B and C, if residue A_i _is aligned to residue B_j _and residue B_j _is aligned to residue C_k_, then this implies that a consistent scoring between A and C should lead to the alignment of A_i _to C_k_. In alignment graph notation consistency implies shifting weights between neighboring edges. Pioneering work on consistency was presented by T-Coffee that implemented consistency transformation referred as triplet library extension. In this transformation the consistency of edge between vertices A_i _and B_j _is increased by iterating all possible C_k _vertices and adding the minimum of (A_i_, C_k_) and (B_j_, C_k_) weights to the (A_i_, B_j_) weight. This idea has been extended in the SeqAn::T-Coffee program, which implements T-Coffee algorithm using SeqAn sequence alignment library [8]. As in the original T-Coffee, existing edges are modified by iteration of neighboring triplets. However, this version also introduces new edges where triplet information requires such for consistency. Tests on benchmark suggest that this approach improves alignment quality [8].

Consistency transformation was also approached in a probabilistic framework. For example, in the ProbCons [14] implementation, vertices of the alignment graph are connected by probabilities according to pairwise hidden Markov models (HMMs). These probabilities are then transformed towards a higher degree of consistency by additively combining probabilities of triplet paths trough all possible vertex triplets. This transformation is similar to triplet library extension, but since edges are weighted by a probability function, the contribution of (A_i_, C_k_) and (B_j_, C_k_) edge-pair to the (A_i_, B_j_) edge is equal to the product of the corresponding probabilities. ProbCons allows repeating the consistency transformation several times, in effect extending consistency to sets of four, five or a larger number of vertices.

Consistency and transitivity concepts were unified in MaxFlow program, which was designed to align motifs of remote homologs that have little sequence similarity when compared directly, but can be connected via a path of pairwise alignments [13]. MaxFlow starts with an alignment graph based on a library of PSI-BLAST alignments. This graph is weighted by assigning each vertex pair a consistency score: the number of common neighbors relative to the total number of neighbors for the two vertices in a pair. The graph is then used to align a pair of distant structural homologs. During the alignment residue pairs are weighted using the weakest path link: the maximum over the path scores defined by the weakest consistency score in a path. In comparison with classical methods, MaxFlow demonstrated superior performance in both reliability and coverage of structural motifs aligned.

In addition to the scoring function, alignment quality is largely affected by guiding tree used in the progressive alignment. Our results suggest that single linkage clustering is optimal for this purpose regardless of the benchmark set.

The progressive alignment method has a serious pitfall: subgroups of sequences are aligned independently of one another, which implies that an optimal subalignment produced near the top of the tree can become a source of errors as it is aligned to other subalignments in the later stages [16]. Consistency scoring, discussed earlier, is one way to address this problem; the other is to correct alignment errors in a post processing step called iterative refinement. In one step of iterative refinement MPSA is partitioned horizontally into two subalignments, which are then realigned and the new alignment is kept if it improves scoring function. Iterative methods based on simulated annealing are too slow to be practical [16]. Other methods differ mainly in the way sequences are divided into two groups before being re-aligned [17]. This can be a leave one out [18], random partitioning [19], or tree-dependent restricted partitioning [16]. The last option was shown to be effective in terms of both quality and speed, and a variation of this technique has been adopted by several current methods including MUSCLE [20] and MAFFT [21].

Here, we present a novel MPSA program based on the SeqAn sequence alignment library. Our implementation has a strict modular structure, which allows to swap different components of the alignment process and, thus, to investigate their contribution to the alignment quality. We used our program to see how alignment quality changes depending on the input information, guiding trees, the applied consistency transformations, and the strategy for iterative refinement during post-processing. This is the first time these strategies are comprehensively and clearly compared using a single implementation platform. Our results confirm the existing knowledge on which strategies are efficient. We also show that the best strategy is comparable in accuracy to the best software in the field.

## Results

To compare alignment strategies we systematically varied information sources, guiding trees, consistency, clique transformation and iterative refinement options, evaluating the resulting alignments on BAliBASE and SABmark.

### Comparison of information sources

**Figure 1:**
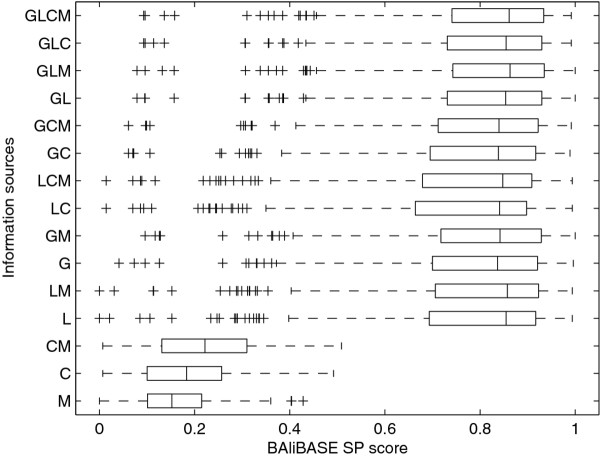
**Performance of strategies with different information sources on BAliBASE**. Boxplots show the sum-of-pairs (SP) score achieved by different strategies for alignments in the BAliBASE benchmark database. Boxplots display first, second and third quartiles as vertical lines; outliers are shown as pluses. The strategies tested differ in the combination of pairwise sequence information that is used to construct the alignment graph. Combinations include: C, longest common subsequence, L, the four top scoring local alignments, G, global alignment, M, GTG motifs.

**Figure 2:**
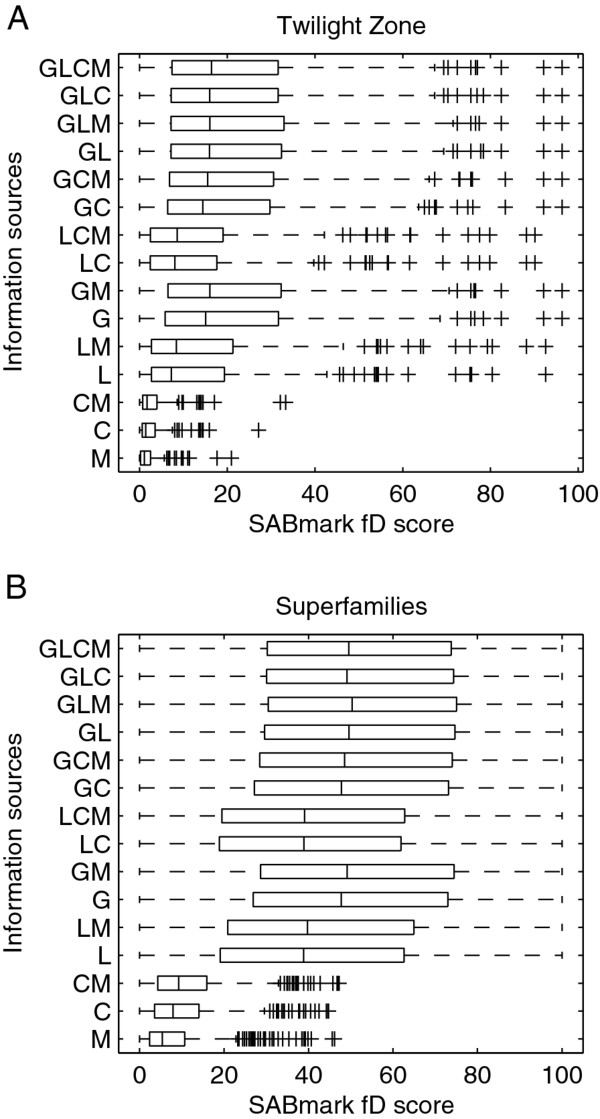
**Performance of strategies with different information sources on SABmark**. Boxplots show developer (FD) score (equivalent to sum-of-pairs [SP] score) achieved by different alignment strategies for (A) "Twilight Zone" and (B) "Superfamily" sets in the SABmark benchmark database. Boxplots display first, second and third quartiles as vertical lines; outliers are shown as pluses. The strategies tested differ in the combination of pairwise sequence information that is used to construct the alignment graph. Combinations include: C, longest common subsequence, L, the four top scoring local alignments, G, global alignment, M, GTG motifs.

We found that using both global and local pairwise alignments to construct the alignment graph, resulted in high quality multiple alignments for both BAliBASE (Figure 1) and SABmark (Figure 2A and 2B) benchmark databases. Adding longest common subsequences to global and local alignments had minor effect on the quality of the multiple alignments. Adding external information in the form of GTG motifs, extracted using motif tracking as described in the original GTG article [22], increased the quality of the multiple alignments by an average of 1% for both BAliBASE and SABmark (full data available in Additional file 2: Tables S2 and S3).

### Guiding trees

We found that single linkage clustering is clearly the best option for all reference sets in BAliBASE and SABmark benchmarks (see Tables 1 and 2). To our surprise, it produced significantly better alignments than commonly used neighbor joining. For BAliBASE, single linkage increased accuracy relative to neighbor joining by 3% and for SABmark benchmark by 5%. We note that this comparison is based on alignments produced with no consistency transformation or postprocessing to account for errors introduced by heuristic nature of progressive alignment. It is probable that when consistency or postprocessing is included the particular type of the guiding tree used becomes less relevant.

**Table 1:** Comparing guiding tree performance on BAliBASE

	RV11 (38)		RV12 (44)		RV20 (41)		RV30 (30)		RV40 (49)		RV50 (16)		Overall (218)		
		
Guiding tree	SP	TC	SP	TC	SP	TC	SP	TC	SP	TC	SP	TC	SP	TC	Time(sec)
NJ	0.554	0.275	0.877	0.664	0.881	0.280	0.798	0.366	0.864	0.413	0.855	0.450	0.805	0.408	23.71

MIN	**0.587**	0.286	**0.908**	**0.772**	**0.902**	**0.357**	**0.832**	**0.457**	**0.884**	**0.483**	**0.871**	**0.540**	**0.831**	**0.483**	24.00

MAX	0.575	0.284	0.901	0.744	0.888	0.313	0.822	0.393	0.850	0.385	0.839	0.389	0.813	0.418	**23.30**

UPGMA	0.585	**0.302**	0.905	0.761	0.891	0.329	0.828	0.431	0.863	0.422	0.859	0.469	0.822	0.452	23.32

WUPGMA	0.583	0.294	0.905	0.761	0.896	0.329	0.829	0.432	0.860	0.423	0.850	0.453	0.821	0.448	23.52

Columns show the average sum-of-pairs (SP) and true-column (TC) scores achieved by different alignment strategies for each of the six BAliBASE references. The number of alignments in each reference is given in parentheses. Overall values for the entire database are reported in addition to the mean execution time of each strategy. The best results in each column are shown in bold. The strategies differ in the algorithm used to build the guiding tree: neighbor joining (NJ), single linkage (MIN), complete linkage (MAX), UPGMA and weighted UPGMA (WUPGMA)

**Table 2:** Comparing guiding tree performance on SABmark

	Twilight zone (209)			Superfamily (425)		
	
Guiding tree	FD	FM	Time(sec)	FD	FM	Time(sec)
NJ	22.63	16.69	0.56	51.26	39.71	0.50
MIN	**23.75**	**17.29**	**0.53**	**53.88**	**41.27**	**0.49**
MAX	23.02	16.83	0.54	52.42	40.31	0.50
UPGMA	23.35	16.97	0.54	53.29	40.91	0.50
WUPGMA	23.42	17.01	0.53	53.31	40.90	0.50

Columns show the average developer (FD) score (equivalent to sum-of-pairs [SP] score), modeller (FM) score and run time achieved by different alignment strategies for the "Superfamily" and "Twilight Zone" sets in the SABmark database. The number of alignments in each reference is given in parentheses. The best results in each column are shown in bold. The alignment strategies differ in the algorithm used to build the guiding tree: neighbor joining (NJ), single linkage (MIN), complete linkage (MAX), UPGMA and weighted UPGMA (WUPGMA)

### Consistency and clique transformations

Our results show that triplet library extension improves alignment accuracy for all sets in both benchmarks when compared to alignments produced with no consistency transformation (referred here as the basic strategy). Triplet_T-Coffee_, which is limited to applying consistency to the existing edges, improved BAliBASE alignments by 1.7%, SABmark Twilight Zone alignments by 3.3% and SABmark Superfamily alignments by 1% (see Tables 3 and 4). Triplet_SeqAn_, which introduces new edges when implied by consistency, was considerably better, enhancing BAliBASE accuracy by 3%, SABmark Superfamily accuracy by 3.1% and SABmark Twilight alignment accuracy by a remarkable 11.2%. Reiterating triplet_SeqAn _transformation for the second time either enhanced or deteriorated alignment accuracy depending on the particular set. The effect was small and has little practical value.

**Table 3:** Comparing score transformation strategies on BAliBASE

	RV11 (38)		RV12 (44)		RV20 (41)		RV30 (30)		RV40 (49)		RV50 (16)		Overall (218)		
		
Strategy	SP	%basic	SP	%basic	SP	%basic	SP	%basic	SP	%basic	SP	%basic	SP	%basic	Time (sec)
basic	0.587	100.0	0.908	100.0	0.902	100.0	0.832	100.0	0.884	100.0	0.871	100.0	0.831	100.0	**24.44**

maxflow	0.585	99.6	0.910	100.2	0.909	100.8	0.836	100.4	0.895	101.2	0.880	100.9	0.836	100.6	25.04

triplet_*T-Coffee*_	0.613	104.4	0.914	100.6	0.914	101.3	0.840	100.9	0.901	101.9	0.888	101.9	0.845	101.7	27.85

triplet_*SeqAn*_	0.642	109.3	0.929	102.3	0.918	101.8	0.839	100.8	**0.911**	**103.1**	0.895	102.7	0.856	103.0	44.83

triplet_*SeqAn *_× 2	**0.648**	**110.3**	0.928	102.2	0.917	101.7	**0.842**	**101.1**	0.910	103.0	**0.896**	**102.8**	**0.857**	**103.1**	608.79

clique	0.530	90.2	0.896	98.7	0.820	90.9	0.614	73.7	0.758	85.8	0.675	77.4	0.715	86.1	1764.83

triplet_*T-Coffee *_+ clique	0.637	108.4	**0.930**	**102.4**	**0.919**	**101.8**	0.839	100.8	0.905	102.4	0.885	101.6	0.852	102.6	1753.63

maxflow + clique	0.590	100.4	0.924	101.8	0.904	100.2	0.819	98.3	0.876	99.1	0.879	100.8	0.832	100.1	2337.67

Columns show the average sum-of-pairs (SP) score and the percentage value of the SP score relative to the basic strategy (%basic) achieved by each alignment strategy for each of the six BAliBASE references. The number of alignments in each reference is given in parentheses. Overall values for the entire database are reported in addition to the mean execution time of each strategy. The best results in each column are shown in bold

**Table 4:** Comparing score transformation strategies on SABmark

Strategy	Twilight zone (209)			Superfamily (425)		
	
	FD	% basic	Time (sec)	FD	% basic	Time (sec)
basic	24.05	100.0	**1.10**	53.74	100.0	0.95
triplet_*T-Coffee*_	24.83	103.3	1.10	54.30	101.0	**0.90**
triplet_*SeqAn*_	**26.74**	**111.2**	1.97	55.40	103.1	1.60
triplet_*SeqAn *_× 2	25.87	107.6	17.38	**55.45**	**103.2**	14.39
triplet_*SeqAn *_× 3	20.78	86.4	77.65	50.77	94.5	68.59
clique	20.05	83.4	7.63	48.66	90.5	6.35
triplet_*T-Coffee *_+ clique	25.28	105.1	9.54	54.65	101.7	7.97
triplet_*SeqAn *_+ clique	25.36	105.4	11.55	54.46	101.3	7.90
maxflow + clique	21.82	90.7	10.34	52.02	96.8	8.70

Columns show the average developer (FD) score, the percentage value of the FD score relative to the basic strategy (%basic) and the average run time achieved by different alignment strategies for the "Superfamily" and "Twilight Zone" sets in the SABmark database. The number of sequences in each reference is given in parentheses. The best results in each column are shown in bold

Applying clique transformation decreased alignment accuracy when compared to the basic strategy. Applying clique transformation after the graph has been made consistent by triplet_T-Coffee _generally provided an additional improvement relative to triplet_T-Coffee_. However, this is not practical since this transformation multiplied the required computational time by a factor of 8 for SABmark sets and by a factor of 65 for the BAliBASE sets. This is due to the huge amount of edges generated by the clique transformation which makes the progressive alignment step computationally demanding. The task appeared even intractable when applying clique transformation after triplet_SeqAn _consistency for some of the BAliBASE alignments, for which reason we do not report on these results here. Running clique transformation after triplet_SeqAn _consistency for SABmark alignments decreased alignment accuracy. This suggests that triplet_SeqAn _is sufficient to introduce edges that have practical utility for the MPSA. Combining MaxFlow consistency with clique transformation had minor improvement on BAliBASE alignments and deteriorated quality of SABmark alignments.

### Iterative refinement

All iterative refinement strategies improved alignment accuracy, but the tree dependent strategies were more efficient. It made little difference, whether the partitioning was done randomly (tree_Random_) or systematically in breath-first order (tree_BF_). Random partitioning improved accuracy of BAliBASE alignments by 1%, tree_Random _by 2.6% and tree_BF _by 2.8%, relative to alignments produced with no refinement (see Table 5). Improvement of accuracy for SABmark Twilight alignments were 5.9%, 9% and 8.5%, respectively; and for SABmark Superfamily alignments 1.6%, 3.2% and 2.9%, respectively (Table 6). Interestingly, the tree dependent strategies are comparable in time and accuracy to the triplet_SeqAn_, which was the best consistency transformation as presented above. The mean SP score for BAliBASE alignments produced with triplet_SeqAn _was 0.856, the corresponding score for tree_BF _was very similar: 0.854. Computation times for these two strategies were also comparable: triplet_SeqAn _took on average 45 seconds and tree_BF _on average 54 seconds per BAliBASE alignment. Moreover, applying tree_BF _iterative refinement on BAliBASE alignments produced under triplet_SeqAn _consistency had no effect on accuracy when measured up to three decimal points of the SP score (Table 5). The same tendency can be seen from SABmark alignments: differences between triplet_SeqAn _and tree_BF _accuracy are rather cosmetic and tree_BF _is on average slightly faster (1.2 sec per alignments against two seconds per alignment for Twilight alignments and one sec against 1.6 sec for Superfamily alignments). For this benchmark, however, applying tree_BF _refinement to alignments produced under triplet_SeqAn _did result in some improvement (Table 6).

**Table 5:** Iterative refinement versus consistency, compared on BAliBASE

Strategy	RV11 (38)		RV12 (44)		RV20 (41)		RV30 (30)		RV40 (49)		RV50 (16)		Overall (218)		Time (sec)
		
	SP	%basic	SP	%basic	SP	%basic	SP	%basic	SP	%basic	SP	%basic	SP	%basic	
basic	0.587	100.0	0.908	100.0	0.902	100.0	0.832	100.0	0.884	100.0	0.871	100.0	0.831	100.0	**24.4**

random × 100	0.623	106.1	0.922	101.5	0.902	100.0	0.832	100.0	0.885	100.1	0.871	100.0	0.839	101.0	40.6

tree_*Random *_× 100	0.625	106.4	0.929	102.3	0.918	101.7	**0.846**	**101.6**	0.909	102.8	0.890	102.2	0.853	102.6	39.4

tree_*BFS *_× 100	0.626	106.5	0.927	102.1	0.916	101.5	0.846	101.6	**0.912**	**103.2**	**0.898**	**103.1**	0.854	102.8	54.1

triplet_*SeqAn*_	0.642	109.3	0.929	102.3	**0.918**	**101.8**	0.839	100.8	0.911	103.1	0.895	102.7	0.856	103.0	44.8

triplet_*SeqAn*_+ tree_*BFS *_× 100	**0.643**	**109.5**	**0.930**	**102.4**	0.917	101.7	0.842	101.1	0.909	102.8	0.894	102.6	**0.856**	**103.0**	195.4

Columns show the average sum-of-pairs (SP) score and the percentage value of the SP score relative to the basic strategy (%basic) achieved by each alignment strategy for each of the six BAliBASE references. The number of alignments in each reference is given in parentheses. Overall values for the entire database are reported in addition to the mean execution time of each strategy. The best results in each column are shown in bold

**Table 6:** Iterative refinement versus consistency, compared on SABmark

	Twilight zone (209)			Superfamily (425)		
	
Strategy	FD	% basic	Time (sec)	FD	% basic	Time (sec)
basic	24.05	100.0	**1.10**	53.74	100.0	**0.95**
random × 100	25.47	105.9	1.64	54.62	101.6	1.49
tree_*Random *_× 100	26.21	109.0	1.51	55.43	103.2	1.30
tree_*BFS *_× 100	26.08	108.5	1.19	55.29	102.9	0.98
triplet_*SeqAn*_	26.74	111.2	1.97	55.40	103.1	1.60
triplet_*SeqAn *_+ tree_*BFS *_× *100*	**27.39**	**113.9**	7.35	**55.51**	**103.3**	5.75

Columns show the average developer (FD) score, the percentage value of the FD score relative to the basic strategy (%basic) and the average run time achieved by different alignment strategies for the "Superfamily" and "Twilight Zone" sets in the SABmark database. The number of alignments in each reference is given in parentheses. The best results in each column are shown in bold

The relative speed of iterative refinement strategies depended largely on the number of sequences in the alignment. For BAliBASE alignments, which on average contain 30 sequences, run times of random and restricted partitioning were comparable. For SABmark alignments, which on average contain 8 sequences, restricted partitioning was slightly faster. Partitioning alignment along all possible edges in the guiding tree was generally faster than random tree dependent partitioning for alignments with a small to moderate number of sequences (BAliBASE sets RV11, RV12, RV40 and RV50 and both SABmark sets), but became slower as the number of aligned sequences approached the number of refinement iterations (BAliBASE sets BB20 and BB30 that have on average 46 and 63 sequences per alignment, respectively).

### Comparison to other programs

We compared our optimal strategies to the eight leading multiple alignment programs: (1) ProbCons 1.12 [14], (2) T-Coffee 8.98 [10], (3) MUSCLE 3.8 [20], (4) MAFFT 6.847 [21], (5) ClustalW 2.0.12 [6], (6) Clustal Omega 1.0.3 [23], (7) Kalign 2.03 [24] and (8) MSAProbs 0.9.5 [25]. MAFFT was run with L-INS-i option; other programs were run with default parameters. All programs were run with a single core. The comparison was done on BAliBASE (Table 7), SABmark (Table 8) and PREFAB (Table 9). The optimal strategy for BAliBASE alignments, referred as MMSA in Table 7, was to use global and local pairwise alignments complemented with GTG motifs as input information; to apply consistency transformation triplet_SeqAn_; and to align the sequences using a single linkage guiding tree. No postprocessing was performed. For SABmark we chose to test two strategies: the same strategy as for BAliBASE, and another strategy, that was augmented with iterative refinement of type tree_BF _(referred as MMSA::tree_BF _in Table 8). On BAliBASE our aligner ranked fourth in terms of alignment accuracy. MSAProbs, MAFFT and ProbCons were more accurate, while Kalign, MUSCLE, MAFFT and both Clustals had better time performance. Also for SABmark our aligner ranked fourth in term of accuracy, this time it was MSAProbs, ProbCons and T-Coffee that performed better. The fastest were again Kalign, both Clustals, MUSCLE and MAFFT.

**Table 7:** Performance of aligners on the BAliBASE benchmark database

	SP						
	
Aligner	RV11 (38)	RV12 (44)	RV20 (41)	RV30 (30)	RV40(49)	RV50 (16)	Overall (218)
MMSA	0.642	0.929	0.918	0.839	0.911	0.895	0.856
ProbCons	0.670	0.941	0.917	0.845	0.903	0.894	0.862
T-Coffee	0.656	0.939	0.914	0.837	0.893	0.895	0.856
MUSCLE	0.572	0.915	0.889	0.814	0.865	0.835	0.815
MAFFT	0.662	0.935	0.927	**0.868**	**0.926**	0.903	0.870
ClustalW	0.501	0.865	0.852	0.725	0.789	0.742	0.746
ClustalO	0.590	0.906	0.902	0.862	0.902	0.862	0.837
Kalign	0.605	0.912	0.900	0.813	0.884	0.820	0.822
MSAProbs	**0.682**	**0.946**	**0.928**	0.865	0.925	**0.908**	**0.876**

	**Time(sec)**						
	
**Aligner**	**RV11 (38)**	**RV12 (44)**	**RV20 (41)**	**RV30 (30)**	**RV40(49)**	**RV50 (16)**	**Overall (218)**

MMSA	0.817	1.772	69.797	119.424	38.725	38.453	44.831
ProbCons	2.481	7.052	142.641	297.473	108.307	124.235	113.698
T-Coffee	15.058	23.310	259.227	646.212	202.948	210.856	226.269
MUSCLE	0.701	0.824	5.490	7.538	9.519	7.002	5.179
MAFFT	0.840	1.580	14.071	25.811	27.455	18.434	14.698
ClustalW	0.310	0.923	13.281	25.231	8.155	8.982	9.480
ClustalO	0.406	0.747	4.224	4.824	5.214	4.790	3.368
Kalign	**0.054**	**0.090**	**0.435**	**0.562**	**0.559**	**0.411**	**0.352**
MSAProbs	2.092	6.436	141.371	295.506	86.463	102.198	105.678

The values in the upper table show the average sum-of-pairs (SP) score and the values in the lower table mean run time achieved by different aligners for each of the six BAliBASE references. The number of alignments in each reference is given in parentheses. Overall values for the entire database are reported in the last column. The best results in each column are shown in bold

**Table 8:** Performance of aligners on the SABmark benchmark database

	Twilight zone (209)			Superfamily (425)		
	
Aligner	FD	FM	Time(sec)	FD	FM	Time(sec)
MMSA	26.74	19.13	1.97	55.40	42.10	1.60
MMSA::treeBF	27.39	19.45	7.35	55.51	42.07	5.75
ProbCons	28.23	20.65	1.36	56.26	43.26	1.37
T-Coffee	27.75	20.50	2.34	55.86	43.03	2.21
MUSCLE	23.32	16.28	0.34	52.43	39.60	0.36
MAFFT	25.70	18.84	0.70	55.01	42.22	0.55
ClustalW	21.85	14.80	0.21	50.29	37.76	0.21
ClustalO	22.17	16.49	0.22	52.63	40.85	0.17
Kalign	21.87	15.24	**0.06**	49.70	38.02	**0.05**
MSAProbs	**28.57**	**20.86**	1.11	**57.13**	**43.92**	1.16

Columns show the average developer (FD) score, modeller (FM) score and run time achieved by different aligners for the "Twilight Zone" and "Superfamily" sets in the SABmark database. The number of alignments in each reference is given in parentheses. The best results in each column are shown in bold

**Table 9:** Performance of aligners on the PREFAB benchmark

	Identity range (main set)					
		
Aligner	0-0.2 (1104)	0.2-0.4 (400)	0.4-0.7 (109)	0.7-1 (69)	Overall (1682)	Time(sec)
MMSA	0.58	0.88	0.98	0.98	0.69	21.47
ProbCons	0.61	0.90	0.97	0.98	0.72	46.68
T-Coffee	0.60	0.89	0.97	0.98	0.71	110.66
MUSCLE	0.56	0.88	0.97	0.98	0.68	2.00
MAFFT	0.62	0.90	**0.98**	**0.98**	0.72	4.16
ClustalW	0.48	0.84	0.98	0.99	0.62	4.83
ClustalO	0.584	0.895	0.976	0.987	0.700	1.419
Kalign	0.525	0.845	0.977	0.984	0.649	**0.160**
MSAProbs	**0.635**	**0.913**	0.974	0.979	**0.737**	49.192

	**Identity range (weighted set)**					
		
**Aligner**	**0-0.2 (59)**	**0.2-0.4 (30)**	**0.4-0.7 (4)**	**0.7-1 (7)**	**Overall (100)**	**Time(sec)**

MMSA	0.43	0.82	0.96	0.98	0.60	2.82
ProbCons	**0.50**	0.87	0.93	0.99	**0.66**	14.18
T-Coffee	0.46	0.75	0.93	0.99	0.60	33.74
MUSCLE	0.42	0.84	0.95	0.99	0.61	1.32
MAFFT	0.46	0.87	0.96	**0.99**	0.64	1.31
ClustalW	0.39	0.88	0.96	0.99	0.60	1.60
ClustalO	0.488	0.876	0.964	0.989	0.658	1.107
Kalign	0.435	0.876	0.963	0.989	0.627	**0.114**
MSAProbs	0.484	**0.881**	**0.964**	0.990	0.658	12.660

Columns show the average Q (equivalent to SP) score achieved for subsets of PREFAB benchmark database, that correspond to different identity values of the structural pair. The number of alignments in each identity range is given in parenthesis. Overall average scores and run time are given in the last two columns. Values for the main and weighted sets are given separately. The best results in each column are shown in bold

For PREFAB we chose to use the same strategy as for BAliBASE. On the main set this yielded moderate accuracy outperforming only the fast aligners: MUSCLE, Kalign and ClustalW. On the weighted reference set our code performed poorly, since it does not account for the overrepresented families (Table 9).

We conclude that, although our modular implementation of the best alignment strategies does not outperform the best aligners in the field, the performance is at a comparable level.

## Discussion

We have completed a comprehensive comparison of graph based strategies for aligning multiple sequences. Our results suggest clear guidelines for a number of choices made during construction of a multiple protein sequence alignment. First, we showed that pairwise global and local alignments contain sufficient information to construct a high quality multiple alignment. When reliable external information, the GTG motifs in our case, is available, it will most likely improve the accuracy and thus should also be included. Second, single linkage clustering is almost invariably the best algorithm to build a guiding tree for progressive alignment. Third, triplet_SeqAn _is the most efficient consistency transformation from those compared. It can have a large improvement on alignment quality, particularly for alignments in the twilight zone. Alternatively, one can apply tree dependent partitioning as a post processing step, which was shown to be comparable with triplet_SeqAn _consistency transformation in both time and accuracy.

As a more subtle result, we found that transitivity, which in principle can increase the sensitivity of the aligner, will in most cases introduce more noise than signal. When we iterated triplet_SeqAn _two times, in effect transferring edge weights within neighborhoods of four vertices, the quality of the alignments increased slightly. However, when iterations were repeated three times, extending the influenced neighborhood to five vertices, the quality invariably deteriorated. The same was the case for clique transformation, where any two vertices, connected by a path, were connected by an edge and thus were allowed to directly influence each other during the progressive alignment.

## Conclusions

We showed that graph based modular implementation allows to compare the contribution of different algorithmic components to the alignment quality and computation time. We demonstrated that shifting edge information within triplets of alignment graph vertices prior to the progressive step and the tree-dependent iterative refinement after the progressive step are equally effective strategies that significantly improve accuracy, particularly for the case of distant homologs. As a negative result we report that extending the neighborhood of edge shifting to five or more vertices introduces more noise than signal.

## Methods

### Algorithm overview

Our program starts by gathering input information from pairwise alignments into an alignment graph. The graph is then refined, transformed and finally fed to a progressive alignment routine that builds an alignment. The resulting alignment is iteratively refined in a post processing step. Altogether there are six steps, detailed below. Each step was implemented as a combination of SeqAn library methods and our own code. The contribution of SeqAn library is outlined in Additional file 2: Table S1.

#### 1. Computing information sources

Global, local and longest common subsequence alignments were computed using SeqAn library with Blosum62 substitution matrix, gap opening penalty set to -13 and gap extension penalty set to -1. Global alignments were constructed using Gotoh's affine gap cost and local alignments using Smith-Waterman algorithms. From local alignments we selected the best four, possibly overlapping, matches; as our preliminary tests indicate that including a larger number of local matches has little effect on accuracy (data not published). We acknowledge that the selection of substitution matrix and gap penalties will have a large effect on the pairwise alignments and hence on the overall MPSA. The values applied here are based on the literature and other implementations.

Additionally, we compiled a collection of conserved motifs based on the Global Trace Graph (GTG) [22]. These were extracted using motif tracking as described in the original GTG article. Alignments and motifs were converted into edges between aligned sequence segments referred hereafter as segment matches.

#### 2. Initializing the alignment graph

The vertices of the graph represent segments of the sequence in the set of proteins to be aligned. The edges of the graph represent possible alignments between these segments. After the edges are generated in the first step, they are assigned weights using Blosum62 substitution matrix. Blosum62 was offset to include only positive values. After this, the structure of the alignment graph is simplified by refinement. During the refinement segment matches are cut into smaller parts in such a way that none of the matches partly overlap (for details see [8]).

#### 3. Score transformation

Score transformations can both modify the edge weights of existing edges, and create new edges. An overview of score transformations is presented in Additional file 3: Figure S1.

##### 3A. Consistency transformation

Alignment graph is made consistent using either triplet library extension introduced in T-Coffee, triplet_T-Coffee_, a variation of this introduced in SeqAn, triplet_SeqAn_, or the consistency measure used in MaxFlow [13]. Triplet_SeqAn _can be repeated several times, in effect extending consistency to sets of four, five or larger number of vertices.

##### 3B. Clique transformation

Connected components are identified using Kruskal's algorithm and converted to cliques. Kruskal's is used to cluster alignment graph vertices by iterating all edges in the order of decreasing edge weight. This produces a collection of spanning trees, which are used to connect each pair of vertices in a tree with a weight equal to the weakest link of the path connecting the two vertices.

#### 4. Tree construction

When global alignments are included in the list of information sources (see above), they are used to calculate the distance matrix. Otherwise, the distance matrix is calculated using k-mer counting. A guiding tree is constructed from the distance matrix using neighbor joining, single linkage, complete linkage, UPGMA or weighted UPGMA. When the guiding tree is constructed using neighbor joining, the root is placed between the last two taxa to be joined.

#### 5. Progressive alignment

Sequences are progressively aligned in the order defined by the guiding tree. In each step, we apply heaviest common subsequence algorithm to align two subalignments. This is a segment based dynamic programming that finds the maximum weight trace from a set of refined alignment segments.

#### 6. Iterative refinement

We implemented three different refinement strategies: random partitioning introduced by Berget & Munson [19], and the two tree-dependent restricted partitioning strategies referred as tree_Random _and tree_BF_. Tree_Random _is the same strategy as used by MUSCLE and MAFFT: sequences are partitioned by cutting a random edge of the guiding tree. Tree_BF _was designed to be a time efficient version of tree_Random_: each edge of the tree is cut only once in the breath-first order.

### Testing methodology

We run our program with options corresponding to each of the tested strategies on BAliBASE 3.0 [26] and SABmark 1.65 [27] alignment benchmark databases. The best strategy was also tested on PREFAB 4.0 [20] benchmark. Tests were performed on a 2.93 GHz Intel Xeon X7350 with 66 GB RAM.

The BAliBASE 3.0 benchmark is a collection of 218 reference protein alignments, compiled from FSSP structural alignments [28], HOMSTRAD [29] databases and hand constructed alignments. The database is organized into five reference sets: in Reference 1 sequences are equidistant and have two levels of conservation; in References 2 a highly divergent "orphan" is aligned with a family of close homologs; in Reference 3 subgroups with < 25% residue identity between groups are aligned; Reference 4 contains sequences with large N/C-terminal extensions; and Reference 5 sequences with large insertions in the middle of the aligned blocks. Alignments were scored relative to the core blocks of benchmark multiple alignments using quality measures proposed by BAliBASE: the sum of pairs score (SP) and the true column score (TC). SP is equal to the number of residue pairs correctly aligned in the evaluated alignment relative to the total number of residue pairs in the benchmark alignment. TC is the number of columns with correct alignment divided by the total number of columns in the benchmark alignment.

The SABmark 1.65 benchmark contains pairwise structural alignments from SOFI [30] and CE [31] databases, that are organized according to SCOP classification. There are two sets: the "Twilight Zone" set contains 1740 domains grouped into 209 SCOP folds, and the "Superfamilies" set that contains 3280 domains grouped into 425 SCOP superfamilies. Sequences in each of the twilight subsets are restricted to have at most 25% and those in superfamilies subsets to have at most 50% pairwise identity. The quality of SABmark alignments was evaluated using FD and FM scores based on reference structural alignments. The FD score measures the number of correct residue pairs relative to the number of paired residues in the reference alignment and FM score relates the number of correct pairs to the total number of paired residues in the test alignment.

PREFAB 4.0 is a large database generated automatically by supplementing structural pairs from FSSP database with homologs found through PSI-BLAST queries. Each alignment set is filtered to have at most 80% identity and limited to a set of 50 random homologs. There are 1682 alignments in the main set and 100 alignments in the weighted set. The weighted set consists of families with one highly over-represented sub-family. The accuracy of the multiple alignment for a given PREFAB alignment set is evaluated relative to the consensus of FSSP and CE alignments of the corresponding structural pair. Alignments were evaluated using PREFAB Q score, which is the analog of BAliBASE SP and SABmark FD scores.

## Competing interests

The authors declare that they have no competing interests.

## Authors' contributions

IP conceived of the study, carried out implementation, testing and benchmark validation, and drafted the manuscript. LH participated in the design and coordination of the study and helped to draft the manuscript. All authors read and approved the final manuscript.

## Supplementary Material

Additional file 1 **Source code, Makefile, installation instructions and test alignments**. Click here for file

Additional file 2 **Table S1**. Outline of MMSA implementation. This table presents the main alignment steps and the corresponding components and functions. Some functions are referred in the main text using abbreviations listed in the fourth column. Source code for any function can be found in one of the two packages: SeqAn sequence alignment library or MMSA code deposited at http://ekhidna.biocenter.helsinki.fi/MMSA. **Table S2**. Performance of strategies with different information sources on the BAliBASE benchmark. Columns show the average sum-of-pairs (SP) and true-column (TC) scores achieved by different alignment strategies for each of the six BAliBASE references. The number of sequences in each reference is given in parentheses. Mean values for the entire database are reported in addition to the mean execution time of each strategy. The best results in each column are shown in bold. The strategies tested differ in the combination of pairwise sequence information that is used to construct the alignment graph. Combinations include: C, longest common subsequence, L, the four top scoring local alignments, G, global alignment, M, GTG motifs. **Table S3**. Performance of strategies with different information sources on the SABmark benchmark Columns show the average developer (FD) score (equivalent to sum-of-pairs [SP] score) and modeller (FM) score achieved by different alignment strategies for the "Superfamily" and "Twilight Zone" sets in the SABmark database. The number of sequences in each reference is given in parentheses. Mean execution time of each strategy is reported in seconds. The best results in each column are shown in bold. The strategies tested differ in the combination of pairwise sequence information that is used to construct the alignment graph. Combinations include: C, longest common subsequence, L, the four top scoring local alignments, G, global alignment, M, GTG motifs. Click here for file

Additional file 3 **Figure S1**. Overview of the score transformations. A) Alignment graph of four sequences after refinement has resolved conflicts between segment matches. B) Applying triplet_T-Coffee _reinforces connections within triplet cliques. C) Applying triplet_SeqAn _reinforces connections within triplet cliques and introduces new edges between vertices that have a common neighbor. Note that edges are never introduced between vertices that belong to the same sequence. D) Applying MaxFlow consistency followed by a clique transformation: edges within spanning trees are weighted by the relative number of common neighbors, and new edges are introduced to convert each spanning tree into a clique (or almost a clique, since edges are never introduced between vertices that belong to the same sequence). Click here for file
