# Supplementary material for: Comprehensive comparison of graph based multiple protein sequence alignment strategies
Source: BMC Bioinformatics. 2012 Apr 29;13:64. doi: 10.1186/1471-2105-13-64 (PMC3375188; doi:10.1186/1471-2105-13-64)
Supplement: Additional file 2 — Table S1. Outline of MMSA implementation. This table presents the main alignment steps and the corresponding components and functions. Some functions are referred in the main text using abbreviations listed in the fourth column. Source code for any function can be found in one of the two packages: SeqAn sequence alignment library or MMSA code deposited at http://ekhidna.biocenter.helsinki.fi/MMSA. Table S2. Performance of strategies with different information sources on the BAliBASE benchmark. Columns show the average sum-of-pairs (SP) and true-column (TC) scores achieved by different alignment strategies for each of the six BAliBASE references. The number of sequences in each reference is given in parentheses. Mean values for the entire database are reported in addition to the mean execution time of each strategy. The best results in each column are shown in bold. The strategies tested differ in the combination of pairwise sequence information that is used to construct the alignment graph. Combinations include: C, longest common subsequence, L, the four top scoring local alignments, G, global alignment, M, GTG motifs. Table S3. Performance of strategies with different information sources on the SABmark benchmark Columns show the average developer (FD) score (equivalent to sum-of-pairs [SP] score) and modeller (FM) score achieved by different alignment strategies for the "Superfamily" and "Twilight Zone" sets in the SABmark database. The number of sequences in each reference is given in parentheses. Mean execution time of each strategy is reported in seconds. The best results in each column are shown in bold. The strategies tested differ in the combination of pairwise sequence information that is used to construct the alignment graph. Combinations include: C, longest common subsequence, L, the four top scoring local alignments, G, global alignment, M, GTG motifs. [file 1471-2105-13-64-S2.DOC]

# Supplementary tables

#

### Supplementary Table1 – Outline of MMSA implementation

This table presents the main alignment steps and the corresponding components and functions. Some functions are referred in the main text using abbreviations listed in the fourth column. Source code for any function can be found in one of the two packages: SeqAn sequence alignment library or MMSA code deposited at <http://ekhidna.biocenter.helsinki.fi/MMSA>.

##

### Supplementary Table 2 - Performance of strategies with different information sources on the BAliBASE benchmark

Columns show the average sum-of-pairs (SP) and true-column (TC) scores achieved by different alignment strategies for each of the six BAliBASE references. The number of sequences in each reference is given in parentheses. Mean values for the entire database are reported in addition to the mean execution time of each strategy. The best results in each column are shown in bold. The strategies tested differ in the combination of pairwise sequence information that is used to construct the alignment graph. Combinations include: C, longest common subsequence, L, the four top scoring local alignments, G, global alignment, M, GTG motifs.

##

### Supplementary Table 3 - Performance of strategies with different information sources on the SABmark benchmark

Columns show the average developer (FD) score (equivalent to sum-of-pairs [SP] score) and modeller (FM) score achieved by different alignment strategies for the “Superfamily” and “Twilight Zone” sets in the SABmark database. The number of sequences in each reference is given in parentheses. Mean execution time of each strategy is reported in seconds. The best results in each column are shown in bold. The strategies tested differ in the combination of pairwise sequence information that is used to construct the alignment graph. Combinations include: C, longest common subsequence, L, the four top scoring local alignments, G, global alignment, M, GTG motifs.
