# Supplementary material for: Comprehensive comparison of graph based multiple protein sequence alignment strategies
Source: BMC Bioinformatics. 2012 Apr 29;13:64. doi: 10.1186/1471-2105-13-64 (PMC3375188; doi:10.1186/1471-2105-13-64)
Supplement: Additional file 3 — Figure S1. Overview of the score transformations. A) Alignment graph of four sequences after refinement has resolved conflicts between segment matches. B) Applying tripletT-Coffee reinforces connections within triplet cliques. C) Applying tripletSeqAn reinforces connections within triplet cliques and introduces new edges between vertices that have a common neighbor. Note that edges are never introduced between vertices that belong to the same sequence. D) Applying MaxFlow consistency followed by a clique transformation: edges within spanning trees are weighted by the relative number of common neighbors, and new edges are introduced to convert each spanning tree into a clique (or almost a clique, since edges are never introduced between vertices that belong to the same sequence). [file 1471-2105-13-64-S3.DOC]

# Supplementary figures

### Supplementary Figure1 – overview of the score transformations

A) Alignment graph of four sequences after refinement has resolved conflicts between segment matches. B) Applying triplet_T-Coffee_ reinforces connections within triplet cliques. C) Applying triplet_SeqAn_ reinforces connections within triplet cliques and introduces new edges between vertices that have a common neighbor. Note that edges are never introduced between vertices that belong to the same sequence. D) Applying MaxFlow consistency followed by a clique transformation: edges within spanning trees are weighted by the relative number of common neighbors, and new edges are introduced to convert each spanning tree into a clique (or almost a clique, since edges are never introduced between vertices that belong to the same sequence).
